# Protective and Healing Effects of Ghrelin and Risk of Cancer in the Digestive System

**DOI:** 10.3390/ijms221910571

**Published:** 2021-09-29

**Authors:** Grzegorz Ginter, Piotr Ceranowicz, Zygmunt Warzecha

**Affiliations:** Department of Physiology, Faculty of Medicine, Jagiellonian University Medical College, Grzegórzecka 16 St., 31-531 Kraków, Poland; grzegorz.ginter@uj.edu.pl (G.G.); mpwarzec@cyf-kr.edu.pl (Z.W.)

**Keywords:** ghrelin, anti-inflammatory effects, pretreatment, protection, healing, risk of cancer, cachexia

## Abstract

Ghrelin is an endogenous ligand for the ghrelin receptor, previously known as the growth hormone secretagogue receptor. This hormone is mainly produced by endocrine cells present in the gastric mucosa. The ghrelin-producing cells are also present in other organs of the body, mainly in the digestive system, but in much smaller amount. Ghrelin exhibits a broad spectrum of physiological effects, such as stimulation of growth hormone secretion, gastric secretion, gastrointestinal motility, and food intake, as well as regulation of glucose homeostasis and bone formation, and inhibition of inflammatory processes. This review summarizes the recent findings concerning animal and human data showing protective and therapeutic effects of ghrelin in the gut, and also presents the role of growth hormone and insulin-like growth factor-1 in these effects. In addition, the current data on the possible influence of ghrelin on the carcinogenesis, its importance in predicting the risk of developing gastrointestinal malignances, as well as the potential usefulness of ghrelin in the treatment of cancer, have been presented.

## 1. Ghrelin and Its Synthesis

Ghrelin, a 28-amino acid peptide, was primary isolated by Kojima et al. from rat and human stomachs in 1999 [1,2,3]. The main source of endogenous ghrelin in the body is the stomach [1,4]. Ghrelin is created from its 117-amino acid precursor, preproghrelin, which consists of a 23-amino acid signal sequence and the 94-amino acid proghrelin [1,5]. The proghrelin is further converted into acyl-ghrelin, des-acyl ghrelin, and obestatin [5,6,7].

Most studies show that the majority of ghrelin in circulation exists in the form of des-acyl ghrelin [8,9,10]. On the other hand, Blatnik et al. [11] postulate that these observations are a result of errors in sampling, handling, collection, and assessment of serum ghrelin. Blatnik et al. analyzed the acyl ghrelin plasma stability by LC-MS/MS and revealed that acyl ghrelin is enzymatically and chemically converted to des-acyl ghrelin in the presence of active serine proteases and HCl. They concluded that that normally all circulating ghrelin is acylated, and des-acyl ghrelin should not be detectible in healthy human plasma under optimal sample handling and assaying conditions [11].

Acyl-ghrelin is considered to be an active form of this hormone [6,8,12]. Acylation is necessary to stimulate the growth hormone secretagogue receptor (GHSR-1a), currently known as the ghrelin receptor [13]. The ghrelin receptors are mainly expressed in the pituitary gland and hypothalamus, but were also present in other tissues and organs [5,13,14,15]. Expression of ghrelin receptor is highly sensitive to the level of growth hormone. In growth hormone-deficient animals, expression of mRNA for ghrelin receptor is increased. On the other hand, an increase in serum growth hormone level reduces the expression of ghrelin receptor [16].

Acylation of ghrelin is catalyzed by the ghrelin O-acyltransferase (GOAT), which was discovered in 2008 [17]. GOAT belongs to a family of hydrophobic membrane-bound acyltransferases [17,18]. Des-acyl ghrelin does not bind to ghrelin receptor, GHSR-1a, and is deprived of growth hormone releasing activity. However, this form of ghrelin may exhibit some non-endocrinological activity, such as the protection of endothelial cells and cardiomyocytes in the heart, regulation of food intake, gastric and pancreatic secretion, gut motility, adipogenesis, stimulation of bone formation, insulin secretion, and prevention of skeletal muscle atrophy [2,3,19,20].

Acyl-ghrelin acting on ghrelin receptor (previously known as GHSR-1a) strongly and dose-dependently stimulates synthesis and release of growth hormone in the anterior lobe of the pituitary gland [1,3]. This effect of ghrelin is mainly related to direct stimulation of somatotropes. However, ghrelin also stimulates the liberation of growth hormone via an indirect pathway. Ghrelin, acting on neurons expressing growth hormone-releasing hormone (GH-RH) in the hypothalamus, leads to the secretion of GH-RH by these neurons. Subsequently, GH-RH reaches somatotropes in the anterior part of the pituitary and stimulates them to release the growth hormone [21]. The ghrelin receptor is a G-protein-coupled receptor and signals via a Gq/11 alpha-subunit, that results in the activation of phospholipase C and the synthesis of inositol triphosphate (IP3), and releases Ca2+ from the endoplasmic reticulum [12,22]. On the other hand, Ge et al. [23] have reported that stimulatory effect of ghrelin on ghrelin receptor can be reduced by liver-expressed antimicrobial peptide 2 (LEAP2), an endogenous antagonist of ghrelin receptor. LEAP2 is produced in the liver and small intestine. This peptide inhibits ghrelin receptor activation by ghrelin, leading to reduction in the major effects of ghrelin in the body, such as food intake, growth hormone release, and maintenance of viable glucose levels during fasting. Secretion of endogenous LEAP2 is suppressed by food restriction, and this effect leads to increased reactivity of ghrelin receptor to the action of ghrelin [23]. Moreover, studies performed on neoplastic cell lines suggest that ghrelin may activate P13K/GTP-Rac [24], GHSR/P13K/Akt [25], and GHSR/CaMKII/AMPK/NFκB [26] signaling pathways.

Apart from ghrelin receptor, there is another type of growth hormone secretagogue receptor, GHSR-1b, but this receptor seems to be not biologically active. Its role is unknown [3].

Ghrelin is mainly synthesized in the gastric oxyntic mucosa, but its presence was also found in the oral cavity, small and large bowel, pancreas, thyroid, lung, testis, myocardium, kidney, brain cortex, brain stem, pituitary, hypothalamus, and immune cells [14,15,27,28]. In rats and dogs, ghrelin is produced in the stomach by the neuroendocrine X/A-like cells [29,30]. These cells are small and round. They have no contact with a stomach lumen. In the human stomach, ghrelin is produced in endocrine cells called P/D1 cells. In the small and large bowel, there are two types of ghrelin-secreting cells: closed-type cells with triangular and elongated shapes, and opened-type cells with their apical cytoplasmic process contacting to the intestinal lumen [1,30]. In the pancreas, ghrelin is produced by endocrine and exocrine cells [15,31,32].

In the case of a decrease in the production of ghrelin in the gastric mucosa, a compensatory increase in the production of this peptide in other areas of the body may occur. Partial resection of gastric mucosa, as a result of bariatric surgery leads to a decrease in serum ghrelin level in the early postoperative period [33]. Later, however, this level returns to the initial value [33] or may be even higher than before the operation [34]. In line with those observation are findings of animal studies performed by Camacho-Ramirez et al. [35], who found that a severe reduction in gastric secretion of ghrelin leads to an increase in the islet ghrelin-secreting epsilon cell population, leading to a subsequent recovery of basal serum ghrelin levels.

## 2. Physiological Action of Ghrelin

The main physiological function of ghrelin is dose-dependent stimulation of growth hormone release from the pituitary gland [1,2]. The endocrine effects of ghrelin also include the stimulation of prolactin, cortisol, and adrenocorticotropic hormone secretion [36,37].

**Figure 1:**
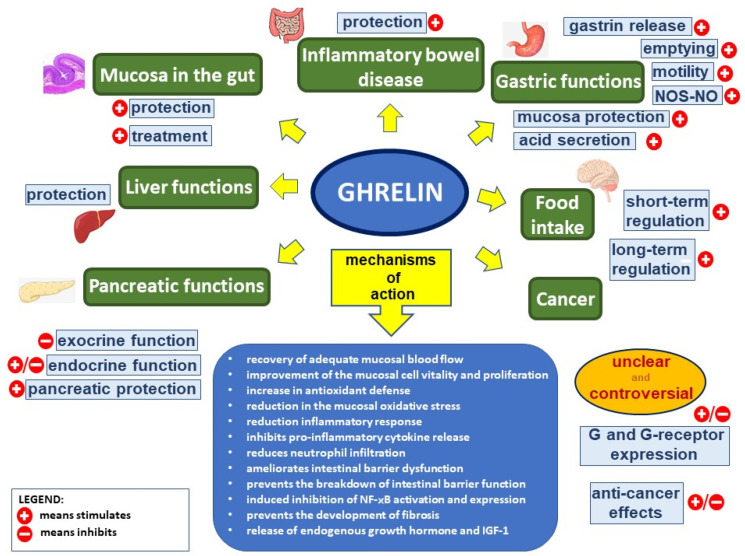
Ghrelin’s effect in the digestive system. Figure legend: NOS–NO—nitric oxide synthase–nitric oxide, G—ghrelin, NF-κB—nuclear factor kappa-light-chain-enhancer of activated B cells, and IGF-1—insulin-like growth factor-1; (+) means stimulates, (−) means inhibits.

Ghrelin is responsible for a positive energy balance. This hormone increases food intake and fat deposition [2,38,39]. The increase in appetite, known as orexigenic effect, is mediated by stimulation of hypothalamic neurons releasing neuropeptide Y, orexin, and agouti-related protein (AgRP), as well as by inhibition of hypothalamic proopiomelanocortin (POMC) neurons [40,41,42]. Among orexigenic peptides stimulating appetite, ghrelin is the only one acting peripherally, whereas all other orexigenic peptides are acting centrally [12]. Besides the stimulation of food intake, ghrelin promotes carbohydrate oxidation and inhibits fat utilization, leading to positive energy balance [43]. The plasma level of ghrelin is negatively correlated with BMI and food intake. For this reason, the plasma concentration of ghrelin is enhanced by anorexia nervosa, starvation, and cachexia, while obesity leads to the opposite effect [44]. Food intake decreases the plasma ghrelin levels, however the degree of this reduction depends on the type of nutrients present in the food. The strongest effect is observed after protein consumption, smaller in case of carbohydrates, and the smallest after the ingestion of lipids [45] (Figure 1).

Ghrelin stimulates gastric motility and gastric emptying [2,46,47]. Impact of ghrelin on the exocrine secretory activity in the stomach is unclear. Gastric acid secretion is dose-dependently increased by the ghrelin administrated peripherally, through a mechanism involving vagal nerve activity and histamine release [46,48,49,50]. Ghrelin effects on gastric acid secretion are in synergy with effects of gastrin [12,51,52]. On the other hand, ghrelin administrated centrally exhibits the opposite effect, inhibiting gastric acid release [12,53,54].

Circulating ghrelin inhibits pancreatic exocrine secretion. Zhang et al. [55] demonstrated that intravenous administration of ghrelin reduces the 2-deoxy-D-glucose- and cholecystokinin-stimulated pancreatic exocrine secretion in anesthetized rats. Moreover, ghrelin inhibits the potassium-stimulated amylase secretion in isolated pancreatic lobules [2,55]. On the other hand, Sato et al. [56] reported, that intracerebroventricular administration of ghrelin rises pancreatic exocrine secretion in conscious rats, and the mechanism of this effect involves the vagal nerves [2,56]. The effect of ghrelin on pancreatic endocrine secretion was initially unclear. Early studies have shown that ghrelin increases insulin secretion by pancreatic β-cells [44,57,58], while next studies have reported that ghrelin inhibits insulin release in the islets of Langerhans [44,59,60]. Currently, it is commonly accepted that ghrelin inhibits glucose-dependent insulin secretion, acting directly on beta-cells in pancreatic islets [44,61,62]. Physiologically, this mechanism is mainly related to ghrelin expressed in pancreatic islets and released into pancreatic microcirculations. Ghrelin has been shown to inhibit insulin release in mice, rats, and humans. Ghrelin antagonists or genetic blockades of islet-derived ghrelin markedly augment glucose-induced insulin release [63]. Inhibition of glucose-induced insulin secretion by ghrelin involves direct interaction of ghrelin with ghrelin receptor coupled to novel cAMP/TRPM2 (cyclic adenosine monophosphate/transient receptor potential melastatin 2) signaling in β-cells [64]. This β-cell unique ghrelin receptor with insulinostatic signaling largely accounts for the systemic effects of ghrelin on circulating glucose and insulin levels. Activation of ghrelin receptor in β-cells inhibits the glucose-induced cAMP and TRPM2 production, and suppresses the glucose-induced [Ca(2+)](i) increase in the β-cell, leading to inhibition of insulin release by β-cells in pancreatic islets [63,64].

There are other functions of ghrelin that are worth mentioning. Vestergaard et al. demonstrated that acyl-ghrelin infusion increases thirst sensation in humans, without affecting diuresis and renal sodium excretion [65]. Ghrelin has been reported to exhibit antidepressant effects [66]. Moreover, Liu et al. showed that ghrelin promotes neural differentiation of adipose tissue-derived mesenchymal stem cells, through the activation of β-catenin and AKT/mTOR signaling pathways [66,67].

## 3. Protective, Anti-Inflammatory, and Healing Effects of Ghrelin in the Digestive System

Protective and healing effects of ghrelin were found in all parts of the digestive system, from the oral cavity to the colon. The influence of endogenous ghrelin level on functional gastrointestinal disorders is unclear. There are studies suggesting that functional dyspepsia is associated with the higher level of serum acyl- or des-acyl ghrelin; however, there is a similar number of articles suggesting the opposite relationship between serum level of acyl- or des-acyl ghrelin and incidence of functional dyspepsia [68].

### 3.1. The Oral Cavity

Ghrelin is synthesized and released by parotid and submandibular salivary glands. Its presence was found in the cytoplasm of striated, intercalated, and excretory ducts, as well as in serous acini of these glands [69]. Ghrelin is also produced and/or present in teeth, taste buds of the tongue, and gingival epithelium, as well as fibroblasts in the lamina propria [70,71,72]. Ghrelin seems to be involved in the tooth development [73]. The concentration of ghrelin in saliva in similar or even higher than that in plasma or serum, the highest level of ghrelin is observed in gingival crevicular fluid [71,74,75]. Proinflammatory factors, such interleukin-1β, increase the expression of mRNA for ghrelin receptor and production of ghrelin receptor in periodontal cells [76]. On the other hand, exogenous ghrelin inhibits the production and release of proinflammatory interleukin-8 by oral epithelial cells stimulated by tumor necrosis factor-α (TNF-α) or lipopolysaccharides [71]. These findings suggest that ghrelin may be involved in endogenous protective mechanisms limiting local inflammation. Moreover, previous experimental studies showed that intraperitoneal administration of exogenous ghrelin significantly accelerate the healing of acetic acid-induced oral ulcers, and that that effect occurs in rats with intact salivary glands, as well in sialoadenectomized rats. The beneficial effect of ghrelin is associated with a reduction in mucosal IL-1β concentration and an improvement of mucosal blood flow, cell vitality, and proliferation. These finding have been confirmed and extended by studies performed on animals [77].

### 3.2. The Esophagus

Clinical and experimental studies have shown the expression of ghrelin receptor to be increased in Barrett’s mucosa in comparison with normal esophageal squamous epithelium. However, ghrelin administration is without any effect on apoptosis of Barrett adenocarcinoma cell line, OE-19 in vitro. On the other hand, administration of ghrelin seems to inhibit Barrett’s carcinogenesis due to suppression of expression of proinflammatory response [78].

Thomas et al. performed a clinical study concerning the relationship between serum level of ghrelin and Barrett’s esophagus [79]. They found that higher ghrelin concentration is associated with an increased risk of Barrett’s esophagus in comparison to the control population, but not when compared with patients with gastroesophageal reflux disease (GERD). Moreover, they reported that ghrelin concentration is associated with the frequency of GERD symptoms.

On the other hand, there is a group of articles showing the influence of ghrelin administration on the inflammatory response in patients with esophageal cancer treated with esophagectomy. Esophagectomy is a highly invasive procedure leading to extended systemic inflammatory response syndrome (SIRS). Continuous infusion of ghrelin (0.5 μg/kg/h) for 5 days after esophagectomy led to a reduction in SIRS duration and lower C-reactive protein and interleukin-6 levels in comparison to the placebo group. Moreover, ghrelin reduced the incidence of pulmonary complications and the time of the negative nitrogen balance during postoperative period [80]. The effect of continuous infusion of ghrelin for 5 days after esophagectomy led to the better therapeutic effect than intermittent infusion for 10 days [81]. In addition, an early drop in plasma level of ghrelin after esophagectomy may be used as a predictor of prolonged SIRS in postoperative period [82].

Moreover, low level of ghrelin seems to be recognized as a risk factor for the development of esophagogastric junctional and gastric adenocarcinomas [83].

The influence of treatment with ghrelin on the healing of esophageal injury is unknown.

### 3.3. The Stomach

Gastroprotective effect of ghrelin was shown in different experimental models of gastric ulcers. In 2003, Sibilia et al. showed that central, as well as peripheral administration of ghrelin inhibits the development of ethanol-induced gastric ulcers in rats [84]. The protective effects of ghrelin given centrally were found to be much more pronounced than the effects of ghrelin given peripherally. Pretreatment with nitric oxide synthases inhibitor, N(omega)-nitro-L-arginine methyl ester (L-NAME), or deactivation of sensory nerves by neurotoxic dose of capsaicin abolished the protective effects of ghrelin given centrally. Sibilia et al. concluded that mechanisms of the gastroprotective effects of ghrelin involve nitric oxide (NO) release and activity of sensory nerve fibers [84]. Similar gastroprotective effect of ghrelin in ethanol-induced gastric ulcer was found by Konturek et al. [85]. Intraperitoneal pretreatment with of ghrelin led to dose-dependent inhibition of the development of gastric lesions, and this effect was associated with the improvement of gastric blood flow and reversion of ethanol-induced increase in TNF-α expression in gastric mucosa. Indomethacin administered prior to the induction of ulcers increased gastric mucosa damage and reduced gastroprotective effects of ghrelin [85].

Ghrelin also exhibits a gastroprotective effect in other models of gastric mucosa damage. Pretreatment with ghrelin inhibits the development of gastric ulcers evoked by water immersion and restrain stress (WRS) [86,87], gastric ischemia followed by reperfusion [88,89], intragastric administration of concentrated hydrochloric acid [90] or alendronate [91].

In addition to its protective effect, ghrelin also exhibits the healing effect in the stomach. Administration of ghrelin after induction of gastric ulcers accelerates the healing of gastric ulcers induced by ethanol [92] and acetic acid [93].

### 3.4. The Small Intestine

Ghrelin has been found to protect the small intestine against damage evoked by ischemia/reperfusion [94,95] and this effect was observed after intracerebroventricular, as well as intravenous, administration of ghrelin. This protective effect was found as reduction in proinflammatory cytokine release and neutrophil infiltration in the intestine and lungs. In addition, ghrelin ameliorated intestinal barrier dysfunction, attenuated intestinal and pulmonary injury, and improved the survival of animals subjected to the gut ischemia/reperfusion-induced damage [94]. Previous studies have also reported that the protective effect of ghrelin against intestinal injury is related to improved intestinal blood flow [95] and promoting the activation of the mTOR/p70S6K signaling pathway [96]. Moreover, intravenous administration of ghrelin receptor antagonist increased the ischemia/reperfusion-induced intestinal and pulmonary injury and animal mortality [94]. This last observation indicates that endogenous ghrelin is involved in maintaining the integrity of the small intestine.

Administration of ghrelin also inhibits the development of experimental damage in the small intestine induced by whole body irradiation [97] and attenuates intestinal barrier dysfunction following intracerebral hemorrhage [98].

Animal experimental studies have also shown that administration of ghrelin exhibits therapeutic effects in injury of the small intestine. Ghrelin accelerates the healing of duodenal ulcers induced by acetic acid [93] or cysteamine [99]. Moreover, ghrelin stimulates intestinal adaptation following a massive resection of the small intestine in parenterally fed rats [100].

### 3.5. The Liver

Previous clinical studies have shown that a low fasting level of ghrelin is associated with increased risk of developing gallstone disease [101], whereas a high fasting serum level of ghrelin reduces the risk of developing nonalcoholic fatty liver disease (NAFLD) [102]. In line with this last observation are the results obtained by Ezquerro et al. [103]. They found that endogenous ghrelin plays a protective role in NAFLD. An increased acylated/desacyl ghrelin ratio in patients with obesity and NAFLD seems to be related to a compensatory mechanism to overcome TNF-α-induced hepatocyte apoptosis, autophagy, and pyroptosis. The protective effects of ghrelin were also shown in animal models of NAFLD. Nagoya et al. have demonstrated that the fatty liver stimulates the autonomic nervous signal circuits, suppressing the progression of the disease by activating the gastric ghrelin expression and the release of IGF-1 from the liver [104]. Moreover, administration of ghrelin in experimental models of NAFLD was found to exhibit preventive and therapeutic effect in this disease [105]. Administration of ghrelin reduced the NAFLD-induced histological changes in the liver, including necrosis, level of apoptotic cells and inflammation foci. This effect was accompanied by a reduction in serum activity of hepatic enzymes, oxidative stress, and lipid peroxidation markers, as well as a decrease in proinflammatory cytokines level. The protective effect of ghrelin on the liver has been also shown in numerous animal models of liver injury. Treatment with ghrelin reduces the acetaminophen- [106], bile duct ligation- [107], ischemia/reperfusion- [108], and the carbon tetrachloride-induced liver injury [107,109,110]. The above articles have shown that the hepatoprotective effect of ghrelin is associated with its antioxidant, anti-inflammatory, and antifibrotic effects. Moreover, Arıcı and Cetin [110] have shown that administration of ghrelin reduces the carbon tetrachloride-induced coagulation disorders.

### 3.6. The Pancreas

Ghrelin exhibits a protective and therapeutic effect on the endocrine and exocrine pancreas.

In the endocrine pancreas, acyl- and des-acyl-ghrelin have been also found to promote proliferation and inhibit apoptosis in pancreatic β-cells and human pancreatic islets [111,112]. In addition, des-acyl ghrelin increases islet cell mass and prevents stretozotocin-induced diabetes in newborn rats [113]. A similar protective effect of ghrelin on pancreatic β-cell has been reported by Wang et al. [114]. Exposure of β-cells to palmitate led to a significant increase in β-cells apoptosis. Administration of ghrelin promoted survival and attenuates the palmitate-induced apoptosis in β-cells [114]. An antiapoptotic effect of ghrelin in pancreatic β-cells was also found by other researchers. Diaz-Ganete et al. performed studies on β-cell line and isolated rats’ pancreatic islets. They found ghrelin has no remarkable effect on β-cells in basal condition without presence of noxious factors. However, when β-cells are exposed to proinflammatory cytokines, ghrelin reduces activation of apoptotic mediators and endoplasmic reticulum stress, restores insulin release in response to glucose, and activates cell survival pathways. They suggested that ghrelin could potentially be effective in preventing or slowing the transition from a preclinical to clinical type 1 diabetes by mitigating insulitis-induced β-cell damage [115]. This concept has been supported by further studies performed with animal models of autoimmune diabetes mellitus. Administration of ghrelin before induction of insulitis significantly reduced the development of diabetes, as well as prevented the reduction in the number of β-cells, islet area, islet number, and β-cell proliferation [116].

In the case of the exocrine pancreas, the protective and therapeutic effect of ghrelin is mainly related to the development and course of acute pancreatitis. Animal experimental studies have shown that pretreatment with ghrelin inhibits the development of acute pancreatitis evoked by cerulein [117], pancreatic ischemia with subsequent reperfusion [118] and taurocholate [119,120]. In the case of acute taurocholate-induced pancreatitis, the anti-inflammatory effects of ghrelin were observed not only in the pancreas, but also in the liver and lung [119,120]. The protective effect of ghrelin was also found in cellular models of acute pancreatitis [121,122,123]. On the other hand, inhibition of ghrelin gene expression in pancreatic acinar cells, AR42J cells, results in increased expression of intracellular inflammatory factors after administration of cerulein [124].

As well as the protective effect, exogenous ghrelin was found to exhibit therapeutic effects in experimental acute pancreatitis. Administration of exogenous ghrelin was found to inhibit the inflammatory process and accelerate the recovery in different animal models of this disease, including cerulein- [125] and ischemia/reperfusion-induced acute pancreatitis [126].

There are also clinical studies showing a relationship between endogenous ghrelin and the course of acute pancreatitis in humans. Some reports suggest that serum ghrelin levels may be a prognostic factor in the course of acute pancreatitis. Wang et al. [127] tested serum ghrelin levels in patients with acute pancreatitis. Patients were divided into three groups: patients with (a) mild; (b) moderate severe; and (c) severe acute pancreatitis. On the 1st day of hospitalization, fasting serum ghrelin concentration was significantly lower in patients with pancreatitis in comparison to healthy controls; the serum level of ghrelin also significantly decreased with increasing severity of acute pancreatitis. During the next four days, fasting serum level of ghrelin increased in all groups of patients, but was still lower than in control group. In addition, serum ghrelin was lower in patients with severe acute pancreatitis than in patients with mild or moderate severe acute pancreatitis [127]. A similar initial drop in ghrelin levels with subsequent increase in the course of acute pancreatitis was also found by Panek et al. [128]. Moreover, they concluded that rising serum ghrelin levels in the course of acute pancreatitis may be a marker of recovery and an indicator of the healing process.

On the other hand, there are also articles reporting that ghrelin affects the course of acute pancreatitis and plays an important role in the regulation of inflammatory response [129], but ghrelin serum level is not a useful predictor of the severity of acute pancreatitis [129,130]. The differences in data presented in above-mentioned articles may be the due to the different number of observations, as well as the criteria for collecting the material and methods for determining ghrelin level. However, it should be stated that all of the above articles concerning clinical observation suggest the participation of endogenous ghrelin in anti-inflammatory and regenerative processes in the course of acute pancreatitis.

The role of endogenous ghrelin was also shown in recovery after pancreatic surgery. Sasaki et al. [131] have shown that plasma ghrelin suppression after pancreatoduodenectomy is a useful marker for predicting postoperative complications. This finding is in line with experimental data showing that exogenous ghrelin enhances endocrine and exocrine regeneration of the pancreas after pancreatectomy [132].

### 3.7. The Large Bowel

The relationship between ghrelin and inflammatory bowel diseases is not clear. Previous studies have shown that patients in the acute phase of Crohn’s disease and ulcerative colitis have higher circulating levels of ghrelin than patients in remission or healthy controls [133,134,135]. In addition, ghrelin mRNA and ghrelin receptor mRNA in colonic mucosa are higher in active IBD patients than in healthy control [136,137]. Moreover, in patients with Crohn’s disease, there is significantly higher percentage of ghrelin-positive peripheral blood T cells than healthy in individuals [136].

There are experimental studies showing protective and healing effect of exogenous ghrelin in colitis. Gonzalez-Rey et al. [138] found that treatment with ghrelin significantly ameliorates the severity of the trinitrobenzene sulfonic acid (TNBS)-induced colitis; as well as colitis evoked by dextran sulfate sodium (DSS). The study was carried out on mice. Administration of ghrelin significantly reduced animals’ weight loss, diarrhea, and inflammation, as well as increased the survival rate of the animals. In line with these findings were clinical and experimental studies performed by Konturek et al. [137]. In the clinical study, they found that patients with ulcerative colitis exhibit a significant upregulation of mRNA for ghrelin and tumor necrosis factor-α (TNF-α) in colonic mucosa in comparison to healthy controls. The ratio of mRNA expression for ghrelin was found to be well-correlated with the severity of inflammation and expression of TNF-α. The animal study showed that treatment with ghrelin accelerates the healing of TNBS-induced colitis in rats, and this effect is accompanied by an increase in inducible nitric oxide synthase mRNA expression and synthesis of cyclooxygenase 2 (COX-2) in the colonic mucosa. These findings suggest that endogenous ghrelin may protect and accelerate the healing of inflamed colonic mucosa, and that ghrelin could be useful in the treatment of ulcerative colitis [137]. The therapeutic effect of ghrelin in TNBS-induced colitis was also shown by Zhang et al. [139].

Similar protective and/or therapeutic effects of ghrelin were also found in other experimental models of inflammatory bowel disease (IBD). Pretreatment [140] or treatment [141,142] with ghrelin reduces the severity of colitis evoked by acetic acid enema and accelerates the healing in this model of IBD. Moreover, Ozturk et al. [143] suggested that protective and therapeutic effects of nesfatin-1 in acetic acid-induced colitis in rats involve activation of ghrelin receptors.

The beneficial effect of ghrelin administration was also shown in dextran sodium sulfate (DSS)-induced colitis in rats [144] and mice [139,145]. In addition, Cheng et al. [145] reported that ghrelin prevented the breakdown of intestinal barrier function in DSS-induced colitis by inhibiting the activation of nuclear factor kappa B (NFκB). This observation is supported by the findings of Zhang et al. [139], which show that the beneficial effect of ghrelin in DSS-induce colitis involves the inhibition of intestinal cell apoptosis.

On the other hand, there are some experimental data suggesting the proinflammatory effect of ghrelin in DSS-induced colitis in mice. De Smet et al. [146] carried out their study in two series. In the first series, they induced colitis in ghrelin(+/+) and ghrelin(−/−) mice. In the second series, they induced colitis in non-inbred Swiss mice by adding 3% dextran sodium sulfate (DSS) to drinking water and dividing the animals into two groups to treated intraperitoneally with saline or ghrelin. De Smet et al. found that the signs of the severity of colitis, such as body weight loss, histological signs of colonic damage, and colonic level of myeloperoxidase activity and interleukin-1β, were significantly less pronounced in ghrelin knockout mice compared to ghrelin(+/+) mice. Moreover, they found that 10 days treatment of non-inbred Swiss mice with exogenous ghrelin enhances the severity of colitis and promotes the release of proinflammatory cytokines in the colon. In conclusion, the authors suggested that endogenous and exogenous ghrelin enhances the colonic manifestations of dextran sodium sulfate-induced colitis in mice [146]. A similar effect was observed by Liu et al. [147]. They compared the severity of DSS-induced colitis in wild mice and ghrelin receptor (−/−) mice. They found that a lack of ghrelin receptor significantly attenuated the severity of DSS-induced colitis. The concept of proinflammatory effects of ghrelin in colitis is also supported by Tian et al. [148]. They reported that knockdown of ghrelin-O-acyltransferase, an enzyme necessary for the production of active, acylated form of ghrelin, attenuates DSS-induce colitis in mice.

The discrepancy between the therapeutic effect of ghrelin in colitis observed by most authors and the harmful effects of ghrelin presented in the last three articles can be explained by the specificity of colitis induced by DSS administered in drinking water. It should be recognized that the severity of colitis most likely depends on the total amount of DSS taken, as well as the amount of DSS taken per unit of body mass. On the other hand, ghrelin increases food [38] and water [65] intake. This most likely causes the amount of DSS ingested to increase. Thus, the greater damage to the colon in animals with active ghrelin receptors, preserved ghrelin production capacity, and receiving exogenous ghrelin is most likely not a result of the damaging effects of ghrelin, but of the increased intake of DSS.

## 4. Mechanisms of Protective and Therapeutic Effects of Ghrelin in the Digestive System

### 4.1. Anti-Inflammatory Effects

Anti-inflammatory effects play an essential role in protective and therapeutic effect of ghrelin in the gut. Ghrelin exhibits anti-inflammatory effects by the inhibition of proinflammatory processes and the stimulation of anti-inflammatory processes [149]. Numerous experimental studies indicate that administration of ghrelin inhibits the expression, synthesis, and release of proinflammatory cytokines such as interleukin-1β (IL-1β), IL-6, IL-8, and tumor necrosis factor-α (TNF-α). This effect was observed in all parts of the digestive system, including the oral cavity [71,77,150], esophagus [80,81], stomach [91,92], liver [106], pancreas [117,118,125], and colon [142]. IL-1-β is a well-known mediator of inflammation and plays an essential role in the induction of local and systemic acute phase response, and in the release of other factors of the proinflammatory cytokine cascade [151,152,153,154]. IL-1-beta acts directly, but also induces the release of other proinflammatory factors such as IL-6, TNF-α, and prostaglandins [151,153,155].

The development and progress of inflammation is related to the activation of the nuclear factor-κB (NF-κB) proinflammatory signaling pathway [156]. The binding of IL-1β and TNF-α to IL-1 and TNF receptor, respectively, as well as pathogen-associated molecular patterns (PAMPs) or damage-associated molecular patterns (DAMPs) to pattern recognition receptors (PRRs), especially Toll-like receptors (TLRs), leads to the activation of downstream transcription factor NF-κB and mitogen-activated protein kinase (MAPK) [149,156]. NF-κB is a protein complex, including, NF-κB2 p52/p100, NF-κB1 p50/p105, c-Rel, RelA/p65, and RelB. In an activated state, NF-κB is located in the cytosol in complexes with inhibitory IκB proteins. The PAMPs, DAMPs, proinflammatory cytokines, and antigen receptors activate an IKK complex (IKKβ, IKKα, and NEMO), leading to activation of IκB kinase (IKK). Next, IKK phosphorylates IκBα protein, resulting in dissociation of IκBα from NF-κB. Active NF-κB complexes translocate into the nucleus and induce the expression of target genes [149].

Previous studies showed that ghrelin inhibits the translocation of NF-κB into the nucleus, leading to suppression of proinflammatory cytokine production [120,145,149,157].

The anti-inflammatory effect of ghrelin is also related to the inhibition of mitogen-activated protein kinase (MAPK) signaling. Administration of ghrelin increases expression of MAPK phosphatase-1, an enzyme providing a negative feedback signal to decrease the activity of MAPKs [158,159].

Anti-inflammatory effects of ghrelin were also observed in a histological examination. Ghrelin reduces pancreatic edema, inflammatory leukocyte infiltration, and vacuolization of acinar cells in all models of acute pancreatitis and hemorrhages [117,118,120,125]. A similar reduction in inflammatory leukocytic infiltration in animals treated with ghrelin was found in experimental models of colitis [140,141]. Activation of leukocytes and release of proinflammatory cytokines are responsible for local pancreatic damage and development of systemic inflammatory response syndrome (SIRS) and multiple organ failure [160].

In agreement with the ghrelin-evoked reduction in serum, tissue level of proinflammatory cytokines, and tissue inflammatory infiltration, is the reduction in tissue activity of myeloperoxidase. Myeloperoxidase is a peroxidase enzyme most abundantly expressed in neutrophil granulocytes. Myeloperoxidase catalyzes production of hypochlorous acid and tyrosyl radicals; those factors have a strong antibacterial and antiviral effect. However, free radicals, apart from a protective effect against infectious factors, have a damaging influence on body cells, leading to destruction of protein, DNA, and lipids [161]. The level of tissue myeloperoxidase activity is closely related to the degree of tissue inflammatory infiltration by neutrophils, and ghrelin-evoked reduction in tissue activity is another indicator of anti-inflammatory activity of this peptide.

### 4.2. Antioxidative Effects

Oxidative stress was originally defined as the imbalance between pro-oxidants and antioxidants in biological systems, and is a result of increased production of reactive oxygen species (ROS) and/or impaired antioxidant capacity [162]. Oxidative stress is one of the causes of the tissue damage in digestive system [163]. Tissue concentration of malondialdehyde (MDA) is a biological marker of oxidative stress. Glutathione peroxidase, superoxide dismutase (SOD), and catalase are the main enzymes responsible for reactive oxygen species neutralization [164]. Oxidative stress is involved in inducing tissue damage in the digestive system [165,166]. Induction of tissue damage increases the concentration of MDA, which is associated with a reduction in tissue SOD activity. Treatment with ghrelin partly but significantly reverses these changes, leading to a reduction in tissue level of MDA and an increase in tissue activity of SOD [91,99,105,109,140,144].

In addition, data presented in chapter 4.1. indicate that ghrelin reduces inflammatory leukocyte infiltration and activity of myeloperoxidase [91,109,141]. This is another mechanism of the antioxidant activity of ghrelin.

The data mentioned above also indicate that ghrelin decreases the oxidative stress in gastrointestinal mucosa, leading to a reduction in tissue damage and the acceleration of organ regeneration.

### 4.3. Antiapoptotic and Proproliferative Effects

Apoptosis, also known as programmed cell death, is a genetically controlled process occurring naturally during development, but can also act as a defense mechanism if cells are damaged or in response to immune reactions [167,168]. There are two distinct apoptotic pathways: extrinsic, or death receptor pathway, and intrinsic, or mitochondrial pathway; simultaneous activation of both pathways potentiates the apoptotic effect [169]. Both pathways activate caspases, leading to permeabilization of the mitochondrial membrane, chromatin condensation, and DNA fragmentation, with the final result in the form of cell death [170,171,172].

The influence of ghrelin on apoptosis in the digestive tract is unclear. There are studies showing that administration of ghrelin inhibits apoptosis in the gastrointestinal tract, although opposite results in different cell types have also been reported. In animal studies, the antiapoptotic effect of ghrelin was observed, among others, by Park et al. [173]. They have found that administration of ghrelin suppresses intestinal apoptosis in fasting rats in a dose-dependent manner. Similar effects were observed by de Segura et al. [174]. They reported that administration of ghrelin reverses gut mucosal hypotrophy-evoked by feeding with an elemental diet. The elemental diet, containing readily absorbable simple nutrients, induces intestinal hypotrophy characterized by decreased proliferation in the ileum and increased apoptosis in jejunum and ileum. Ghrelin administration restored cell proliferation in the ileum and reduced apoptosis in the jejunum to a level observed in normally fed animals. In the ileum, the reversal of the reduction in apoptosis after ghrelin was only partial. Additionally, studies by Ercan et al. showed that ghrelin has antiapoptotic effect in the gut [175]. Administration of ghrelin significantly reversed the sodium metabisulfite-induced elevation of total oxidants status, number of apoptotic cells and caspase-3 expression in gastric mucosa. These effects of ghrelin were additionally associated with an improvement of total antioxidant status and Ki67 expression, a cell proliferation index, in gastric mucosa exposed to sodium metabisulfite. The data mentioned in this paragraph indicate that ghrelin can reverse the damage-induced increase in apoptosis in animals exposed to harmful agents, but the level of apoptosis after administration of ghrelin does not increase above that observed in control healthy animals; it can only reach its value.

A similar effect of ghrelin on apoptosis was observed in studies carried out on normal, non-neoplastic cells. Slomiany and Slomiany reported that ghrelin protection against lipopolysaccharide-induced gastric mucosal cell apoptosis involves constitutive nitric oxide synthase-mediated caspase-3 S-nitrosylation [176]. Ghrelin was also found to protect salivary gland acinar cells against *Porphyromonas gingivalis*-induced apoptosis [177].

There are conflicting data regarding the effect of ghrelin in the digestive tract on apoptosis in cancer cell lines. Ghrelin may act as either antiapoptotic or proapoptotic factors in different cancer cell lines, suggesting that these effects may be dependent on cell type or methodological differences. For example, He et al. reported that ghrelin inhibits 5-fluorouracil-induced apoptosis in HT-29 colon cancer cells by reducing caspase-3 activation and increasing BCL-2/Bax ratio [178]. The opposite effects were observed by Bonfili et al. [179]. They found that ghrelin induces apoptosis in colon adenocarcinoma cells by inhibiting the ubiquitin-proteasome system and by activating autophagy, whereas Konturek et al. reported that ghrelin remained without affecting the apoptosis in Barrett’s adenocarcinoma cell line [78].

Mucosa in the digestive tract is constantly exposed to mechanical, chemical, and thermal injures, as well as to the action of infectious agents. Even under physiological conditions, minor injuries and inflammatory infiltration of the mucosa is observed. In order to prevent mucosa injury and accelerate the healing of mucosal damage, the gastrointestinal mucosa exhibits a high cell proliferation rate similar to that seen in the bone marrow. In humans, mucosal cell renewal takes 4 to 6 days [180].

Studies performed on rats indicate that influence of ghrelin on organ growth in digestive system depends on the age of animals and maturity of hormonal axis: ghrelin-growth hormone-insulin-like growth factor-1 (IGF-1) [181,182]. In young suckling rats, treatment with ghrelin increase serum levels of growth hormone, but is without effect on serum concentration of IGF-1. In these rats, administration of ghrelin was without effect on body weight, whereas pancreatic weight, cell proliferation, and content of amylase were reduced. On the other hand, administration of ghrelin in peripubertal rats leads to significant increase in serum growth hormone and IGF-1, and these effects are associated with significant increase in daily food intake, pancreatic weight, cell proliferation, and content of amylase [181]. Similar effects of ghrelin administration and the relationship between animals age, release of growth hormone and IGF, and organ growth, were observed in the case of the stomach [182]. In turn, in young mature rats, administration of ghrelin increases food intake and leads to an increase in body weight, stimulation of cell proliferation in the stomach and duodenum, and an increase in the weight of these organs, but at the same time may inhibit the production of digestive enzymes [183]. These findings indicate that the effects of ghrelin administration on food intake and organ growth may vary in consecutive periods of life. This conclusion is additionally supported by the observations of Saito et al. [184]. They found that ghrelin inhibits food intake in neonatal chicks. There are also differences in the regulation of endogenous ghrelin secretion in childhood. In prepubertal children, ghrelin secretion is refractory to the inhibitory effect of feeding [185].

Cell proliferation plays an essential role in the protection and regeneration of the gastrointestinal tract. Induction of mucosa damage by various harmful factors in animal models leads to a decrease in mucosa cell proliferation monitored by measurement of DNA synthesis in the digestive tract [166,186,187]. The same effect is also observed in other parts of the digestive system in studies performed in animal models [188,189].

Administration of ghrelin prior to mucosal damage or after them leads to the reversion of damage-induced inhibition of cell proliferation, prevents tissue damage, and/or accelerates mucosa healing [77,93,99,140,141], as well other tissues in the digestive system [117,126].

### 4.4. Improvement of Blood Flow

The digestive system is especially sensitive to hypoxia and a reduction in visceral blood flow. Mucosal blood flow plays an important role in the protection and healing of mucosa in the gastrointestinal tract [190,191,192,193]. Previous experimental studies have shown that exposure of gastric mucosa to potentially noxious factors results in little or no damage, as long as adequate blood flow is maintained [191]. Blood flow protects gastric mucosa from damage by supplying it with oxygen, bicarbonate, and nutritious substances, and removing CO2, hydrogen ions, and other metabolic products, as well as toxic agents diffusing into the stomach wall from the lumen of this organ [190,191,194]. Previous studies have shown that gastric injury is associated with a reduction in mucosal blood flow [195], whereas the protection and healing of gastric mucosa is associated with an increase in gastric blood flow [92,196,197].

The same protective and healing effects of blood flow occurs in other organs of the gut, such as the oral cavity [77], duodenum [93,99], and large bowel [198,199]. In the pancreas, clinical [200,201] and experimental [202,203,204,205,206] studies show that a decrease in pancreatic blood flow always aggravates pancreatic damage, whereas the improvement of pancreatic blood flow reduces the severity of AP.

Numerous previous experimental studies showed that the protective and therapeutic effects of ghrelin in the digestive system are associated with the improvement of organ blood flow. This effect was found, among others, in the oral cavity [77], stomach [92,93], duodenum [93,99], pancreas [117,125,126], and colon [140,141,144].

### 4.5. Direct or Indirect Effects of Ghrelin

Numerous studies, presented in earlier chapters of this article, have shown that treatment with ghrelin produces protective and healing effects in the digestive system. However, an important question arises as to whether these effects are due to the direct action of ghrelin on its receptors located on cells in organs where these effects are observed, or whether they are indirect effects, initiated by the release of growth hormone from the pituitary. The concept that the effects of ghrelin administration are related to the direct action of ghrelin on a target organ is supported by findings that ghrelin receptors are expressed, apart from the pituitary and hypothalamus, in other organs and tissues [5,15], including pancreatic islets and acinar cells [207,208], as well as different immune cells involved in the inflammatory process. The presence of ghrelin receptor was found in human leukemic B, T, and myeloid cell lines, human peripheral lymphocytes and neutrophils [14], and mouse splenic T cells [209,210]. In the case of isolated cells or cell lines, the effects of ghrelin on these cells must be related to the direct action of this hormone on its receptors. For example, the direct effect of ghrelin on its receptor leads to the inhibition of the potassium-stimulated amylase secretion in isolated pancreatic lobules [55]. Additionally, later studies have indicated the ghrelin affects insulin secretion in the isolated islets of Langerhans [59,61,211]. Similarly, ghrelin is able to inhibit exocrine secretion in isolated pancreatic acinar cells [55], as well as apoptosis in β cells [114]. However, even in the case of isolated cells, it is not known whether the activity of these cells is modified in the presence of growth hormone or insulin-like growth facto-1 (IGF-1). The presence of growth hormone receptors has been detected in numerous cells in the digestive system, including pancreatic islets and acinar cells [212,213,214], as well as in immune cells [215]. Additionally, receptors for IGF-1 are present in the endocrine [216] and exocrine [217] pancreas and lymphocytes [218].

On the other hand, there is strong evidence that the biological effects of ghrelin in vivo are mainly indirect effects related to the release of endogenous growth hormone and other hormones in hormonal axis activated by growth factor. The release of growth hormone from the pituitary gland is one of the first effects of ghrelin to be discovered in animals and humans [1]. The concept of an indirect mechanism of protective and therapeutic action of ghrelin in the digestive system is supported by four groups of evidence.

First, the administration of ghrelin stimulates release of GH and IGF-1 in mature individuals [118,182]. Growth hormone is the first step in the GH-IGF-1-hepatocyte growth factor (HGF) hormonal axis [219,220,221].

Secondly, treatment with growth hormone [222,223], IGF-1 [118,224], and HGF [225,226,227] exhibits protective or/and therapeutic effect in experimental acute pancreatitis. These factors were also found to prevent the development of inflammation and damage, and accelerate the healing in the stomach and colon [228,229,230,231,232].

Third, the removal of the pituitary gland lowers serum growth hormone levels below detection, and serum IGF-1 concentration to about 10% of those observed in pituitary intact control animals. In addition, hypophysectomy abolishes the protective and healing effect of treatment with exogenous ghrelin in oral lesions [150], gastric ulcer [93], acute pancreatitis [118,233], and experimental colitis [142].

Fourth, administration of exogenous IGF-1 in hypophysectomized rats increases a concentration of circulating IGF-1 to a level similar to that observed in ghrelin-treated rats with intact pituitary gland, and produces similar protective effects in acute pancreatitis as administration of ghrelin in pituitary-intact animals [118].

## 5. Ghrelin and Cancer

**Figure 2:**
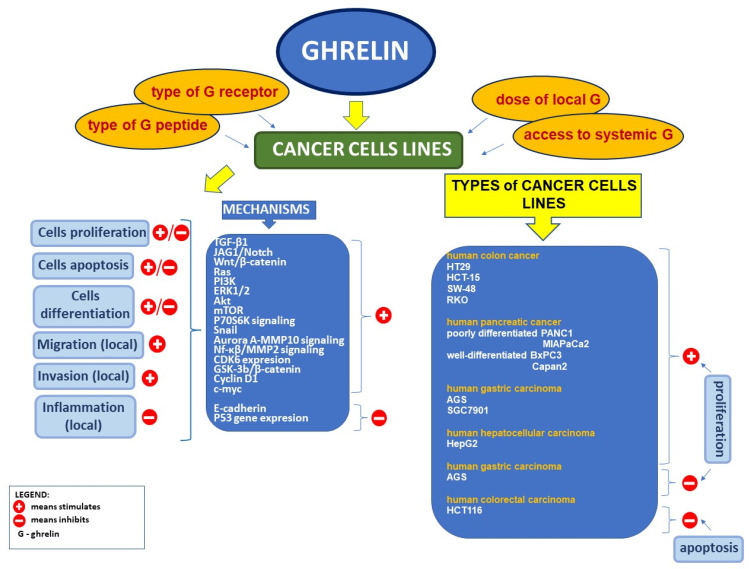
Effects of ghrelin on cancer cell lines.

Reports on the relationship between ghrelin and cancer are unclear and controversial. There are numerous studies showing the expression of ghrelin, and its receptor is observed in many types of cancer cells [234] (Figure 2).

### 5.1. Expression of Ghrelin and Its Receptor in Clinical Neoplasms

Local expression of ghrelin has been observed in range of neoplasms, including, among others, oral, esophageal, gastric, pancreatic, colorectal, breast, ovarian, prostate, thyroid, lung, endometrial, and renal cancer; adrenocortical tumors; pituitary adenomas; and endocrine pancreatic tumors [234,235]. Ghrelin expression was also found in metastatic renal cell carcinoma, and high ghrelin expression is correlated with poor outcome [236]. The presence of ghrelin receptors was shown, among others, in breast, ovarian, and prostate cancers, pituitary tumors, and astrocytoma [237]. Moreover, analysis of cancer genomics data with matched clinical observations suggest that ghrelin might be a critical factor in cancer progression and methastasis [234,238].

On the other hand, the ghrelin is absent in some cases of colorectal cancer, lung cancer, leukemia, and adrenocortical tumors [237].

### 5.2. Effect of Ghrelin on Cell Proliferation and Apoptosis in Tumor Cell Lines

Several factors are involved in cancer development and progression, including cell proliferation, apoptosis, metastasis, angiogenesis, and drug resistance. The effect of ghrelin on cancer cell proliferation is controversial and varies with the type of neoplasm [234]. Several studies have reported that ghrelin promotes neoplasm cell proliferation, including, among others, human colon cancer (HT29 and HCT-15) [239] and (SW-48 and RKO) [240] cell lines; poorly differentiated (PANC1 and MIAPaCa2) and well-differentiated (BxPC3 and Capan2) human pancreatic cancer cell lines [25]; human gastric carcinoma cell lines AGS and SGC7901 [241]; estrogen-independent breast cancer cell lines (MDA-MB-435 and MDA-MB-231) [242]; well-differentiated (Ishikawa), moderately differentiated (HEC1B), and poorly differentiated (KLE) endometrial cancer cell lines [243]; androgen-dependent human prostate cancer LNCaP cell lines [244]; and human hepatocellular carcinoma HepG2 cell lines [245].

In contrast to the abovementioned reports, there are also studies showing that ghrelin may inhibit cancer cell proliferation. Ghrelin-induced inhibitory effects on cell proliferation were observed, among others, in human gastric carcinoma cell line AGS [246]; in N-PAP and ARO thyroid carcinoma cell lines [247]; in human breast carcinoma cell lines (MCF7, T47D, and MDA-MB231) [248]; in ovarian cancer cell line HO-8910 [249,250]; and in human prostate carcinoma PC-3 cells [251].

There are also studies showing that ghrelin is without effect on cell proliferation in estrogen-dependent breast cancer cell lines (T47D and MCF-7) [242].

Furthermore, it should be noted that, even in cancer cell lines in which ghrelin promotes cell proliferation, regulatory mechanisms are retained to prevent ghrelin-induced over-stimulation of these cells. Physiologically, administration of ghrelin increases serum level of growth hormone and IGF-1 [1,220,221]. In turn, an increase in levels of growth hormone [16,252], as well as IGF-1 [253], leads to a statistically significant decrease in mRNA expression for the ghrelin receptor. Moreover, numerous studies indicate that both mRNA and protein expression of ghrelin receptor is downregulated by exposure to ghrelin or synthetic agonists of ghrelin receptor [254,255]. Desensitization of ghrelin receptors is next mechanism protecting cells against receptor overstimulation. Desensitization is a result of a combination of the uncoupling of the receptor heterotrimetric G-proteins and the internalization of cell surface receptors, together with ghrelin to intracellular compartments. Kinetic studies suggest that ghrelin receptor is internalized by endocytosis in time-dependent manner, with a peak at about 20 min after ligand stimulation. After internalization of the ghrelin–ghrelin receptor complex into intracellular vesicles, ghrelin receptor is sorted into the endosomes, and then returned back to the membrane [256,257]. In the case of cancer cell lines, numerous authors, including Lien et al. [239], Duxbury et al. [25], Jeffery et al. [242], and Yeh et al. [244], reported that the administration of ghrelin at submaximal concentrations leads to the highest increase in cell proliferation. On the other hand, the administration of ghrelin at a concentration greater than submaximal leads to a partial reduction in this effect.

Some studies performed on cancer cell lines indicate that ghrelin increases cell migration and invasion [24,25,236,240,241,258,259] and inhibits apoptosis [178,243,244,258,260]. The observation that silencing ghrelin receptor expression inhibits the growth of endometrial cancer cell line is in line with the pro-oncogenic activity of ghrelin. This effect was found in vitro and in vivo studies [261].

On the other hand, there are studies showing that ghrelin exhibits anticancer effects. For example, Hu et al. [246] found that overexpression of ghrelin inhibits gastric cancer cell proliferation, cell migration, invasion, and promotes apoptosis. Those effects are associated with the activation of the AMPK pathway, while treatment with D-[lys3]-GHRP-6, a ghrelin receptor antagonist, reverses these effects, promoting tumorigenesis. The ghrelin-mediated promotion of apoptosis was also reported, among others, in ovarian cancer cell line HO-8910 by Bai et al. [250], in human colorectal carcinoma (HCT116) cell line by Bonfili et al. [179], and in the PC-3 human prostate carcinoma cells by Díaz-Lezama [251]. In addition, ghrelin administration suppresses inflammation-associated colorectal carcinogenesis in mice [262].

### 5.3. Relationship between the Serum Level of Ghrelin and the Risk of Cancer in the Digestive System

**Figure 3:**
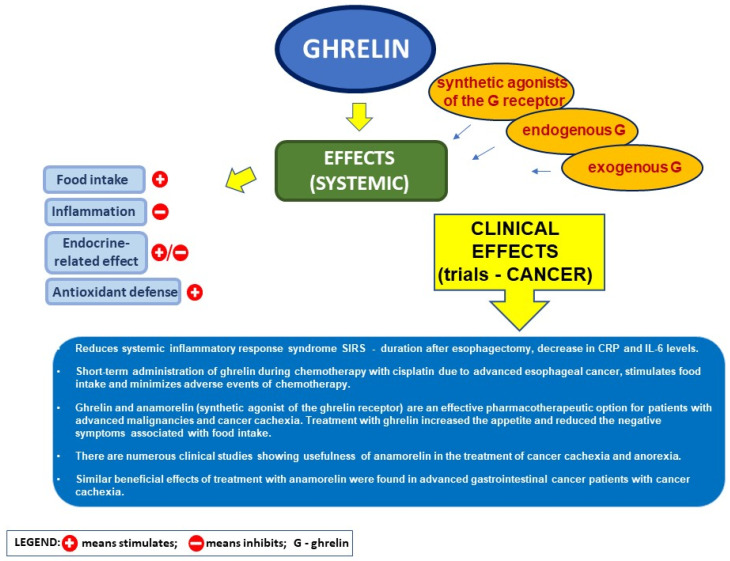
Supportive effect of ghrelin in cancer disease.

There are numerous clinical observations showing the relationship between serum level of ghrelin and incidence of cancers in the digestive system (Figure 3).

A study by Murphy et al. suggests that low baseline serum ghrelin concentration is associated with an increased risk of developing esophageal squamous cell carcinoma, and this relationship is valid for 10 years after blood collection [263]. The similar relationship was found between serum ghrelin and risk of colorectal adenocarcinoma. Low serum ghrelin levels seem to be associated with an increased risk of colorectal cancer [264]. Additionally, in non-cardia gastric adenocarcinoma and gastroesophageal junction adenocarcinoma, low serum ghrelin levels are associated with an increased risk of developing these neoplasms [83]. These observations were confirmed by Pritchett et al. [265] on the basis of the analysis of data collected during the Linxian General Population Nutrition Intervention Trial (NIT) and the Shanghai Women’s Health Study (SWHS). The analysis NIT and SWHS data led to the conclusion that low serum ghrelin concentration is associated with an increased risk of developing gastric cardia adenocarcinoma and non-cardia gastric adenocarcinoma. On the other hand, in contrast to observations obtained by Murphy et al. [263], low ghrelin concentrations at baseline were associated with a reduced risk of developing esophageal squamous cell carcinoma in the NIT. It should be noted, however, that the study by Murphy et al. [263] suggested that high baseline serum ghrelin concentration is associated with a reduced risk of developing esophageal squamous cell carcinoma during 10 years after blood collection. In the case of the studies by Pritchett et al. [265] one-third of esophageal squamous cell carcinoma cases were diagnosed 10 years or later after blood collection and measurement of serum ghrelin concentration, and three-fourths of patients with esophageal squamous cell carcinoma were diagnosed with Helicobacter pylori infection.

The important role of serum ghrelin in the development of colorectal cancer was also confirmed by D’Onghia et al. [266]. They have found serum ghrelin levels are significantly lower in colon cancer patients than in controls. Moreover, serum ghrelin levels decrease in subsequent stages of cancer development [266].

### 5.4. Ghrelin and the Postoperative Course after Heavy Surgery in Cancer Disease

Clinical data indicate that the measurement of serum ghrelin concentration may be useful in prognosis of postoperative courses in patients undergoing esophagectomy with gastric tube reconstruction due to esophageal cancer. Yamamoto et al. [82] have found that a drop in the individual ghrelin ratio in relation to preoperative value (IGR) below 34% on postoperative day 1 is well-correlated with prolonged systemic inflammatory response syndrome (SIRS) after esophagectomy. Similar relationships between plasma ghrelin suppression and early postoperative complications were found by Sasaki et al. in patients after pancreatoduodenectomy [131]. These observations led to a concept that administration of ghrelin may be useful in preventing an early postoperative complication after severe operations. Takata et al. have presented results of a prospective randomized phase II trial conducted to evaluate the efficacy of ghrelin administration in reducing systemic inflammatory response syndrome duration after esophagectomy [80]. They have found that continuous infusion of ghrelin (0.5 μg/kg/h) for 5 days leads to a reduction in SIRS duration and a decrease in CRP and IL-6 levels. In addition, treatment with ghrelin reduced the incidence of pulmonary complications and time of the negative nitrogen balance [80]. Similar beneficial effects of ghrelin infusion in postoperative period in patients with esophageal cancer treated with esophagectomy was observed by Yamashita et al. [81]. Ghrelin also appears to be beneficial in combination with chemotherapy. Short-term administration of ghrelin during chemotherapy with cisplatin due to advanced esophageal cancer stimulates food intake and minimizes adverse events of chemotherapy [267].

### 5.5. Ghrelin and Its Analog in Cancer Cachexia

Approximately 50% of cancer patients exhibit cachexia syndrome, characterized by anorexia and loss of fat and skeletal mass. Cachexia has a huge impact on patients’ quality of life, physical and mental abilities, and sense of dignity. Cachexia is a very serious complication, as the body mass loss in patients with cancer is associated with more frequent and serious chemotherapy-related side effects, fewer chemotherapy cycles completed, a poorer post-operative course, and, most importantly, decreased survival [268]. There are clinical observations indicating that ghrelin and anamorelin are an effective pharmacotherapeutic option for patients with advanced malignancies and cancer cachexia. The study carried out by Blum et al. [269] have shown that natural ghrelin given subcutaneously in advanced cancer patients with cachexia, is safe and well-tolerated without dose-limiting toxicity. In patients’ opinion, treatment with ghrelin increased their appetite and reduced the negative symptoms associated with food intake.

Anamorelin is a non-peptide selective ghrelin receptor agonist that stimulates food intake and exhibits anabolic effects. Anamorelin is administered orally, which makes it easier to use. There are numerous clinical studies showing the usefulness of anamorelin in the treatment of cancer cachexia and anorexia.

In 2015, Garcia et al. published an integrated analysis of two phase 2, randomized, multicenter, placebo-controlled, double-blind trials on effects of treatment with anamorelin in patients with cancer cachexia [270]. Patients were stratified by weight loss severity (5–15%, > 15%) and randomly allocated (1:1) with a computer-generated randomization schedule to anamorelin hydrochloride 50 mg or placebo once-daily for 12 weeks. Forty-four patients were enrolled in the anamorelin group and 38 patients in the placebo group. Over 12 weeks, lean body mass increased in 38 patients in the anamorelin group compared with a decrease in 36 patients in the placebo group. Forty-two (95%) of 44 patients treated with anamorelin and 33 (87%) of 38 patients treated with placebo had adverse events. The most common grade 3–4 adverse events (treatment-related or not) in the anamorelin group were fatigue, asthenia, atrial fibrillation, and dyspnea; in the placebo group, such events were pneumonia, anemia, thrombocytopenia, abdominal pain, anxiety, and dyspnea. Garcia et al. concluded that treatment with anamorelin for 12 weeks has a favorable clinical response profile in patients with cancer anorexia-cachexia syndrome [270].

Talem et al. published an article in 2016 in which they reported the results of two randomized, double-blind, phase 3 trials (ROMANA 1 and ROMANA 2) on the effects of anamorelin administration in patients with inoperable stage III or IV non-small-cell lung cancer and cachexia [271]. In ROMANA 1, 484 patients were enrolled (323 to anamorelin and 161 to placebo), and 495 patients were enrolled in ROMANA 2 (330 to anamorelin and 165 to placebo). The authors found that anamorelin significantly increased lean body mass strength in patients with advanced non-small-cell lung cancer, but not handgrip strength. There were no differences in grade 3–4 treatment-related adverse events between study groups. In both trials, the most common grade 3–4 adverse event was hyperglycemia, occurring in about 1% of patients treated with anamorelin. Talem et al. concluded that, considering the unmet medical need for safe and effective treatments for cachexia, anamorelin might be a treatment option for patients with cancer anorexia and cachexia [271].

Similar beneficial effects of treatment with anamorelin were found, among others, by Takayama et al. [272], Currow et al. [273], and Katakami et al. [274] in patients with non-small cell lung cancer and cachexia; Hamauchi et al. [275] found similar results in advanced gastrointestinal cancer patients with cancer cachexia.

In addition, Malik and Yennurajalingam have suggested that the therapeutic effects of anamorelin in cancer cachexia could be improved via combination of anamorelin with prokinetics such as metoclopramide [276].

## 6. Conclusions

In the gut, ghrelin stimulates food intake, gastric acid secretion, and gastrointestinal motility. Ghrelin exhibits protective and healing-promoting effects in numerous organs, including the digestive system. These effects are due to, among others, the anti-inflammatory and antioxidative properties of ghrelin. In the case of tumors, the effect of ghrelin on their development and course appears to depend on the type of tumor. The results of studies carried out on cancer cell lines are inconclusive. In some cancer cell lines, ghrelin stimulates cell proliferation and inhibits apoptosis, whereas in others exhibits opposite effects. There are studies showing that determining the plasma or serum level of ghrelin may be useful in predicting the risk of cancer development in the digestive system. Clinical data indicate that the measurement of serum ghrelin concentration may be useful in prognosis of postoperative courses in patients undergoing esophagectomy with gastric tube reconstruction due to esophageal cancer, as well as in patients after pancreatoduodenectomy. In addition56, the administration of ghrelin may be useful in preventing an early postoperative complication after severe operations. Infusion of ghrelin led to a reduction in SIRS duration. There are clinical data showing that short-term administration of ghrelin during chemotherapy with cisplatin due to advanced esophageal cancer stimulates food intake and minimizes the adverse events of chemotherapy. There are some reports that a synthetic agonist of the ghrelin receptor, anamorelin, is useful in the treatment of cancer cachexia and anorexia.

## Data Availability

All presented data are in articles shown in the reference list.
